# The Temporal Relation Between Rates of Retinal Nerve Fiber Layer and Minimum Rim Width Changes in Glaucoma

**DOI:** 10.1167/tvst.13.4.10

**Published:** 2024-04-05

**Authors:** Bethany E. Higgins, Hongli Yang, Stuart K. Gardiner

**Affiliations:** 1 Devers Eye Institute, Legacy Health, Portland, OR, USA

**Keywords:** glaucoma, minimum rim width, retinal nerve fiber layer, structural equation model

## Abstract

**Purpose:**

This study aims to determine whether OCT-derived rates of change in minimum rim width (MRW) are associated with and can potentially predict corresponding alterations in retinal nerve fiber layer thickness (RNFLT) in people with glaucoma.

**Methods:**

The rates of change between six-monthly visits were taken from 568 eyes of 278 participants in the P3 Study. Structural equation models (SEM) assessed whether one parameter was predicted by the concurrent or previous rate of the other parameter, after adjusting for its own rate in the previous time interval. Root mean square error of approximation (RMSEA, with 90% confidence intervals [CI]), Tucker Lewis index (TLI) and the comparative fit index (CFI) assessed goodness of fit.

**Results:**

Models without a time lag provided a better fit for the data (RMSEA = 0.101 [CI, 0.089, 0.113]), compared to a model featuring a time lag in RNFLT (RMSEA = 0.114 [CI, 0.102, 0.126]) or MRW (RMSEA = 0.114 [CI, 0.102, 0.127]). The SEMs indicated that rates for both MRW and RNFLT were predicted by their own rate in the previous time interval and by the other measure's change in the concurrent time interval (*P* > 0.001 for all). No evidence of a clinically significant time lag for either parameter was determined.

**Conclusions:**

MRW and RNFLT exhibit concurrent changes over time in patients with glaucoma, with no clinically significant time lag determined.

**Translational Relevance:**

RNFLT may be more useful than MRW in early glaucoma assessment because of its previously reported lower variability and reduced sensitivity to intraocular pressure changes.

## Introduction

Glaucoma manifests as a progressive optic neuropathy marked by characteristic structural alterations in the optic nerve head (ONH) and retinal nerve fiber layer (RNFL), including loss of axons and subsequent functional loss within the visual field (VF).^1^ While functional tests such as VF perimetry have traditionally served as the cornerstone for assessing glaucoma progression, measurement of the structural changes remains key due to better repeatability, objectivity, and quicker acquisition time. Optical coherence tomography (OCT), a widely used tool, is used to monitor glaucomatous changes such as alterations in RNFL thickness (RNFLT), contributing crucial insights into the structural aspect of glaucoma. Indeed, RNFLT has been demonstrated to be directly related to the number of retinal ganglion cell axons that remain.^2^

As the ONH region occupies a pivotal role in glaucoma pathogenesis, measurements taken of the neuroretinal rim such as the minimum rim width (MRW) may provide a more sensitive indicator of early glaucomatous changes. While RNFLT quantifies nerve tissue thickness in the peripapillary region, MRW represents the minimum thickness of nerve tissue surrounding the opening of Bruch's membrane (BMO), averaged around the optic disc (also known as BMO-MRW).^3^^,^^4^

Elevated intraocular pressure (IOP), characteristic of glaucoma, can induce significant changes at the ONH, both a short-term increase in cupping due to the ONH being mechanically displaced posteriorly, and longer-term tissue remodeling, which could enhance the ONH's resistance to increased IOP.^5^ Increased cupping exerts a mechanical influence on the axons, which may result in a reduction of MRW.^6^ Importantly, RNFLT measurement occurs sufficiently outside the cup (typically along a 6° radius circle centered on the BMO centroid), minimizing its susceptibility to these mechanical effects.^7^^,^^8^ This is postulated to be the reason why MRW tends to exhibit greater variability than RNFLT when IOP fluctuates.^9^^–^^11^ In this context, the reduction in MRW due to increased cupping may potentially precede and predict actual axon loss and, therefore, RNFL thinning. Notably, in a nonhuman primate experimental glaucoma model, changes in MRW precede the onset of RNFLT change.^12^ MRW has also been reported to show earlier detectable change than RNFLT in early-stage glaucoma,^3^ and the rate of change of MRW has been found to be consistently greater than RNFLT in patients with early normal-tension glaucoma (NTG).^13^ However, Shi et al.^14^ reported that RNFLT is more likely than MRW to reveal a declining trend over time in patients with central or moderate-to-advanced glaucomatous damage. The potential time lag between MRW and RNFLT alterations underscores the complexity of glaucomatous progression and highlights the need for comprehensive assessments that consider the distinct dynamics of these parameters.

This study investigates whether the rates of change in MRW are associated with and can potentially predict corresponding alterations in RNFLT, situated further away from the critical ONH region, within the context of human glaucoma. Our primary objective is to use structured equation modeling (SEM) to dissect the temporal dynamics of these measures. By rigorously assessing the temporal relationship between MRW and RNFLT changes, we seek to determine the presence or absence of any clinically significant time lag between the two (i.e., greater than six months to ensure it is useful for clinical diagnostics purposes). Such a finding holds implications for our comprehension of glaucoma progression and stands to inform future developments in glaucoma testing.

## Methods

### Participants and Data Collection

Participant data were sourced from the ongoing Portland Progression Project (P3), a longitudinal investigation funded by National Institutes of Health and conducted at the Devers Eye Institute in Portland, Oregon. The P3 study is focused on monitoring progression in individuals with high-risk ocular hypertension and glaucoma who undergo a battery of vision assessments, including standard automated perimetry (SAP) and OCT, approximately every six months. The study adheres to the tenets of the Declaration of Helsinki and complies with the Health Insurance Portability and Accountability Act of 1996. All testing protocols were approved by the Legacy Health Institutional Review Board. All participants provided written informed consent after having the risks and benefits of participation explained to them. Recruitment was conducted by clinicians at Devers Eye Institute, a tertiary eye clinic within Legacy Health; and the inclusion criteria encompassed individuals diagnosed with open-angle glaucoma or those deemed likely to develop glaucoma, as assessed by their attending clinician, to ensure representation of a typical clinical population. Individuals with a history of angle closure, the presence of other ocular pathologies that could potentially impact visual field assessments (e.g., diabetic retinopathy or forms of macular degeneration), those unable to reliably undergo visual field testing, or individuals likely unable to provide high-quality OCT images, were excluded from the study.

Spectral domain OCT testing was performed using a Spectralis OCT (870 nm) instrument (Heidelberg Engineering GmbH, Heidelberg, Germany) with a 6° radius radial scan centered on the ONH. Peripapillary RNFLT is defined as the mean distance between the inner limiting membrane and the outer boundary of the RNFL, measured in micrometers. MRW was calculated as the minimum distance from BMO to the inner limiting membrane, calculated as previously described, based on 24 radial scans centered on the ONH.^15^ Automated layer segmentations were manually corrected by technicians if necessary.^16^ All included OCT scans had a quality score ≥16; quality scores below this were counted as missing data for that visit. Data were only retained if both RNFLT and MRW values were available from scans of high quality conducted in the same session. In addition to OCT imaging, all participants underwent SAP using a Humphrey Field Analyzer II (Carl Zeiss Meditec Inc., Dublin, CA, USA), with the SITA Standard testing strategy and 24-2 test pattern, on the same day. The Glaucoma hemifield test was used for automated evaluation of localized visual field loss occurrences.^17^

Study visits were scheduled approximately every six months. For this analysis, data were binned into six-month time periods (±3 months). The visit within the time period three to nine months after the baseline visit would be Visit 2. If there were more than one visit within the stipulated time period, the first visit was used. When there were multiple scans for the same day, the scans and the associated timestamps were manually checked by the author (BH) to ensure the appropriate scan was selected. After binning the data into these visits, the rate of change between the visits could be calculated. For example, *MRWRate_n_* = *(MRW_n+1_–MRW_n_)/(Date_n+1_–Date_n_)*. The main analysis was conducted on a time series of four rates, using data from five visits, therefore the duration of the data analyzed was approximately two to three years. A secondary analysis was performed using longer time series of 10 rates using data from 11 visits. For this subanalysis the duration of the data analyzed was approximately five to six years.

The longest uninterrupted time series available for the participant was selected for analysis. (For example, if a participant has recorded visits for every six months from 2009 until 2012, missing visits during 2013–2015, but then returns to participate in the study every six months from 2016 until 2018, this analysis would only use data from 2009–2012 as the longest uninterrupted time series available.) If a study visit was missed within the time period, such that *MRW_n+1_* was unavailable, then *MRWRate_n_* was treated as missing data, and *MRWRate_n+1_* = *(MRW_n+2_ – MRW_n_)/(Date_n+2_ – Date_n_)*. This was extended to accommodate up to three consecutive time periods with missing visits.

### Structural Equation Modeling

SEM is a statistical technique used to examine complex relations among multiple variables. It combines elements of factor analysis and regression analysis to provide a comprehensive framework for testing and estimating relationships between observed variables and latent constructs, as well as the relationships among latent constructs themselves.^18^ SEM is well suited for analyzing changes over time because it can accommodate repeated measures of variables and assess how variables change in relation to one another across multiple time points. Furthermore, SEM provides fit indexes to assess how well the proposed model fits the observed data. Researchers can modify and refine their models based on fit indexes, ensuring that the model adequately represents the underlying data structure. SEM has been used previously by Gardiner et al.^19^ to demonstrate that changes in SAP precede and predict changes in RNFLT.

In this study, we are interested in whether there is a clinically significant time lag between *MRWRate_n_* (i.e., the “current” rate, denoted by “_n_”) and *MRWRate_n-1_* (the previous rate, denoted by “_n-__1_”)*;* and *RNFLTRate_n_* and *RNFLTRate_n-1_*, respectively. We will test four SEM models to make these comparisons, which we will refer to as Models A to D.

#### Model A: Current RNFLT Change Predicts MRW Change

$$\begin{array}{c} \begin{array}{c} \mathrm{MRWRat}e_{n}=\mathrm{Intercep}t_{\mathrm{MRW}}+\alpha_{A}\;*\;\mathrm{MBR}\mathrm{Rat}e_{n-1}\\ \;+\;\beta_{A}\;*\;\mathrm{RNFLTRat}e_{n}+\mathrm{Error}\\ \mathrm{RNFLTRat}e_{n}=\mathrm{Intercep}t_{\mathrm{RNFLT}}+\gamma_{A}\\ \;\times \;\mathrm{RNFLTRat}e_{n-1}+\mathrm{Error} \end{array} \end{array}$$

#### Model B: Previous RNFLT Change Predicts MRW Change

$$\begin{array}{c} \begin{array}{c} \mathrm{MRWRat}e_{n}=\mathrm{Intercep}t_{\mathrm{MRW}}+\alpha_{B}\;*\;\mathrm{MBR}\mathrm{Rat}e_{n-1}\\ \;+\;\beta_{B}\;*\;\mathrm{RNFLTRat}e_{n-1}+\mathrm{Error}\\ \mathrm{RNFLTRat}e_{n}=\mathrm{Intercep}t_{\mathrm{RNFLT}}\\ \;+\;\gamma_{B}\,\;\mathrm{RNFLTRat}e_{n-1}+\mathrm{Error} \end{array} \end{array}$$

#### Model C: Current MRW Change Predicts RNFLT Change

$$\begin{array}{c} \begin{array}{c} \mathrm{MRWRat}e_{n}=\mathrm{Intercep}t_{\mathrm{MRW}}+\alpha_{C}\,\;*\;\mathrm{MBR}\mathrm{Rat}e_{n-1}\\ \;+\;\mathrm{Error}\\ \mathrm{RNFLTRat}e_{n}=\mathrm{Intercep}t_{\mathrm{RNFLT}}+\gamma_{C}\;*\;\mathrm{RNFLTRat}e_{n-1}\\ \;+\;\beta_{C}\;*\;\mathrm{MRWRat}e_{n}+\mathrm{Error} \end{array} \end{array}$$

#### Model D: Previous MRW Change Predicts RNFLT Change

$$\begin{array}{c} \begin{array}{c} \mathrm{MRWRat}e_{n}=\mathrm{Intercep}t_{\mathrm{MRW}}+\alpha_{C}\;*\;\mathrm{MBR}\mathrm{Rat}e_{n-1}\\ \;+\;\mathrm{Error}\\ \mathrm{RNFLTRat}e_{n}=\mathrm{Intercep}t_{\mathrm{RNFLT}}\\ \;+\;\gamma_{C}\;*\;\mathrm{RNFLTRat}e_{n-1}\\ \;+\;\beta_{C}*\mathrm{MRWRat}e_{n-1}+\mathrm{Error} \end{array} \end{array}$$

**Figure 1.**
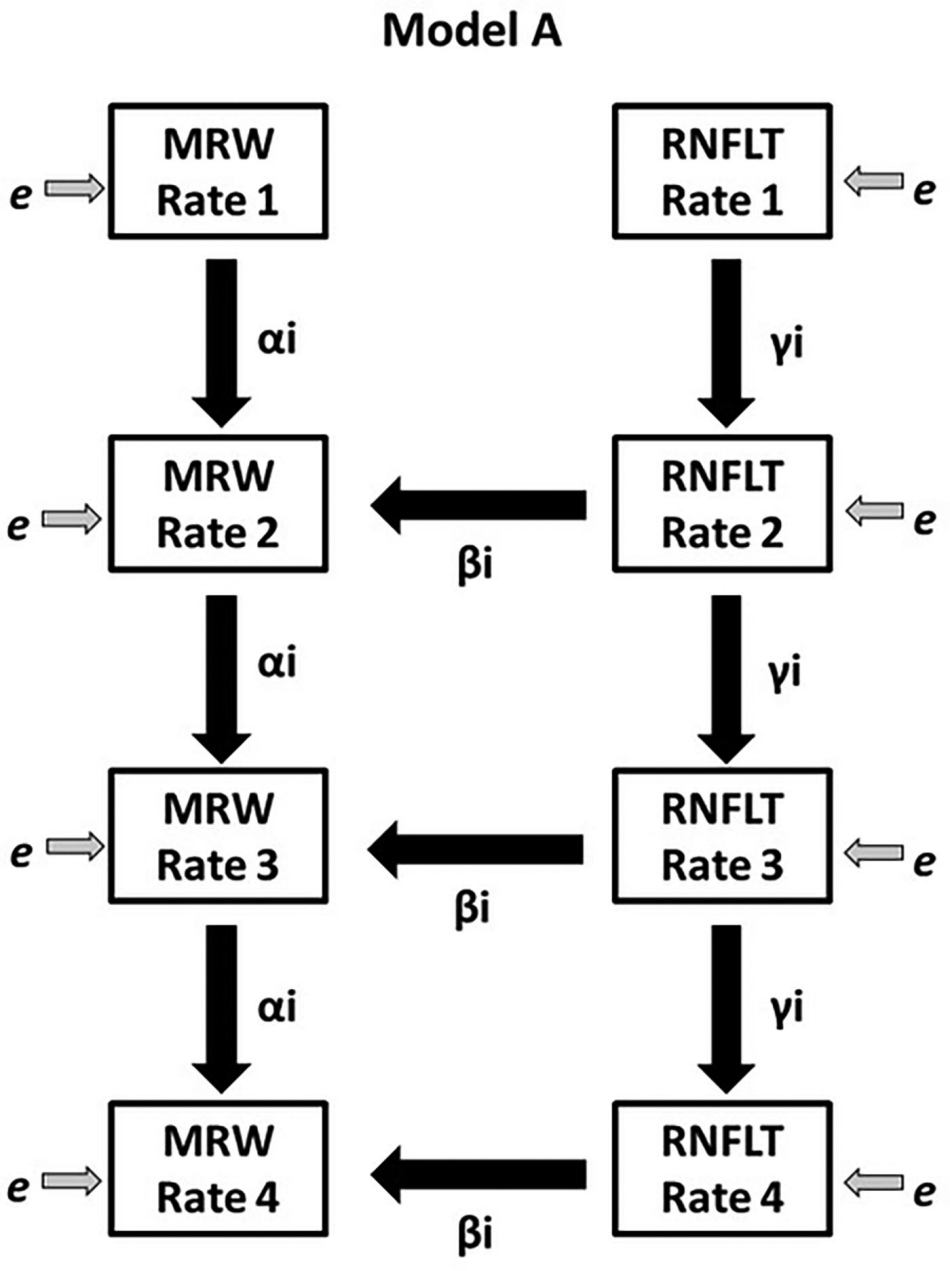
The path diagram for Model A, one of the four SEMs featured in this study, testing if *RNFLTRate_n_* and *MRWRate_n_* (both current rates) predict *MRWRate_n_*_._. The observed variables *RNFLTRate_n_* and *MRWRate_n_* are shown, representing the measured rates of change of the two modalities over period n (from visit n-1 to visit n). *Directional arrows* indicate regressions, with labeled coefficients and measurement errors are illustrated as *e* and are assumed to be Gaussian.

In these models, measurement errors are assumed to be independent identically distributed Gaussian random variables. Coefficients α and γ are always negative; the *ΔMRWRate_n-1_* (i.e., from visit n − 1 to visit n) will be inversely correlated with the *ΔMRWRate_n_* (i.e., from visit n to visit n + 1), because they both have the measurement at visit n in common. We assume that the true rate of axon loss is approximately linear; this is reasonable as the time series are relatively short (five visits, which equates to approximately three years). Figure 1 shows the path diagram for Model A.

The primary hypothesis being tested is that more rapid change in one modality may predict more rapid change in the other modality, either in the same interval or the following interval (i.e., whether β is significantly greater than zero). In other words, if β_A_ is significant, knowing the rate of change of RNFLT improves predictions of the concurrent rate of change of MRW. If β_B_ is significant, the rate of change of RNFLT over the previous time period helps predict the rate of MRW change in the current period; that is, there is a time lag with RNFLT changing earlier than MRW. If β_C_ is significant, knowing the rate of change of MRW improves predictions of the current rate of change of RNFLT. Last, if β_D_ is significant, the rate of change of MRW over the previous time period helps predict the rate of RNFLT change in the current period; that is, there is a time lag with MRW changing earlier than MRW.

Analyses were performed using R statistical software, version 4.0.0, using the lavaan package.^20^ Models were fit using the maximum likelihood estimation because the estimates are unbiased, have the smallest possible variances, and are asymptotically normally distributed, despite the presence of data missingness. Goodness of fit for each model was assessed using the root mean square error of approximation (RMSEA, chosen as our primary measure of goodness of fit because it is an absolute fit index and gives 90% confidence intervals [CI]),^21^ as well as the Tucker Lewis index (TLI)^22^ and the comparative fit index (CFI), both examples of incremental fit indexes.^23^ For context, RMSEA values closer to zero and CFI and TLI values closer to one represent a good fit. A correlational analysis was performed using Spearman's rho, and a Steiger's *Z*-test was used to evaluate differences between two dependent correlation coefficients.

### Subgroup Analysis

To assess the models in people who are glaucoma suspects, subgroups of the patient cohort were analysed: (i) eyes rated both structurally and functionally “normal,” whereby SAP values fell “within normal limits” according to the Glaucoma hemifield test^17^ and OCT structural values within the normative limits^24^ and (ii) eyes rated structurally or functionally “abnormal,” who fell outside the above criteria. To assess the influence of age on ONH deformation, we also assessed a subgroup of patients: (i) aged <65 years and (ii) aged ≥65 years. The SEM-based analysis was repeated in each of these cohorts.

## Results

### Study Population

A total of 568 eyes from 287 people were included in the study. Cohort demographics and clinical characteristics can be found in Tables 1 and 2. Cohort demographics and clinical characteristics for participants in the subgroup analysis can be found in Supplementary Tables S1 and S2. Mean time difference between the first five visits was 215 days, which equates to 6.6 months (standard deviation [SD] ± 79 days; range, 91–772 days). The inter-visit time intervals for intervals where there were no missing visits was 191 days, which equals 6.2 months (SD ± 23 days; range, 118–267 days).

**Table 1.** Demographic Characteristics of n = 287 Participants

Demographics	Number of Participants	Percentage
Gender		
Male	111	39%
Female	176	61%
Ethnicity		
White	259	90.2%
Black	7	2.4%
Asian	12	4.2%
Mixed	4	1.4%
Native American	2	0.7%
Unknown	3	1.1%

Data sourced from self-report.

**Table 2.** Baseline Characteristics of n = 568 Eyes

	Mean	Standard Deviation	Range
Age (years)	64	10.1	33–86
Mean deviation (dB)	−0.9	3.0	−17.7–6.7
Retinal nerve fiber thickness (µm)	85.5	15.2	38.8–121.0
Minimum rim width (µm)	259.0	66.4	79.5–485.5
IOP	17.19	3.71	7–29

Characteristics for the subgroup analyses, in which the cohort was split into eyes inside versus outside normal limits and younger versus older eyes, are presented in Supplementary Table S2.

### Primary Analysis: Does RNFLT Change Predict MRW Change?

The measures of goodness of fit indicated that Model A provides a better fit for the data (RMSEA = 0.101 [CI = 0.089, 0.113]) compared to the Model B (RMSEA = 0.114 [CI = 0.102, 0.126]). Model A has a higher CFI and TLI scores (thus greater improvement in fit over a null model) compared to Model B (see Table 3 for all goodness of fit measures). Notably, there is no overlap in the RMSEA CI between the models, indicating that Model A (current RNFLT change predicts MRW change) yields a statistically significantly better fit than Model B (previous RNFLT change predicts MRW change).

**Table 3.** Goodness-of-Fit Measures for the Models

SEMs	N Eyes	RMSEA	CFI	TLI
Model A	568	0.101	0.628	0.703
(current RNFLTRate predicts MRWRate)		(CI, 0.089–0.113)		
Model B	568	0.114	0.537	0.619
(previous RNFLTRate predicts MRWRate)		(CI, 0.102–0.126)		
Model C	568	0.101	0.629	0.703
(current MRWRate predicts RNFLTRate)		(CI, 0.089–0.113)		
Model D	568	0.114	0.535	0.617
(previous MRWRate predicts RNFLTRate)		(CI, 0.102–0.127)		

RMSEA values closer to zero and CFI and TLI values closer to one indicate a good fit.

**Figure 2.**
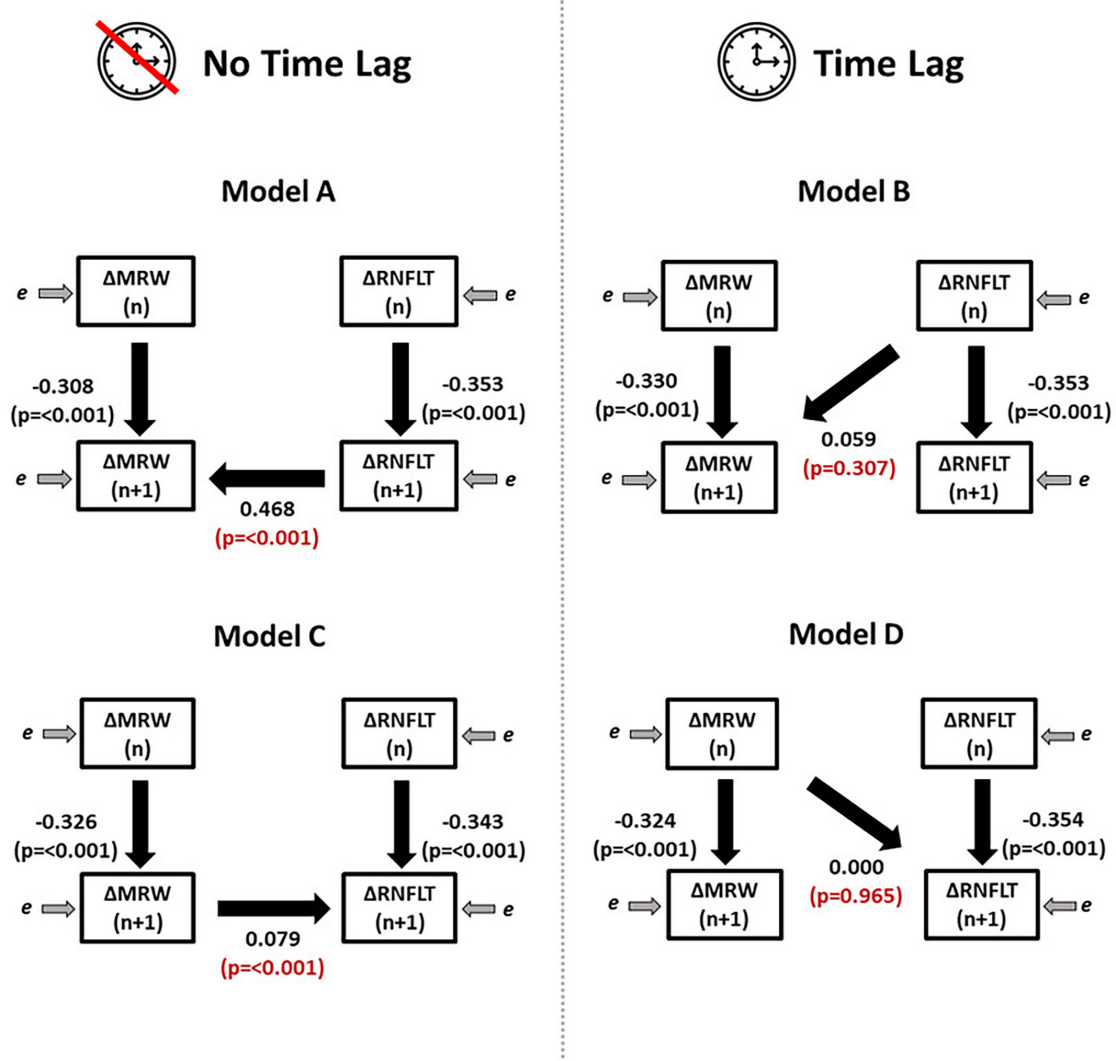
Fitted coefficients for the four SEMs used. Coefficients relating to the observed variables are shown, representing the measured rates of change of RNFLT and of MRW over period n (from visit n to visit n + 1).

Using Model A, we found that *ΔMRWRate_n-1_* and *ΔRNFLTRate_n_* were significant predictors of *ΔMRWRate_n_* (both *P* < 0.001), which is to say that the previous rate of MRW and the concurrent rate of RNFLT significantly predict the current rate of MRW. We did not find evidence that *ΔRNFLTRate_n-1_* significantly predicts *ΔMRWRate_n_*. Therefore no evidence of a clinically significant time lag for RNFLT parameter was determined. The coefficient estimates and the respective significance levels for all four models can be found in Figure 2. The coefficient estimates and intercepts that best fit Model A are as follows:

#### Model A: Current RNFLTRate Predicts MRWRate

$$\begin{array}{c} \begin{array}{c} \Delta MRWRate_{n}=-0.008-0.308*\Delta MRWRate_{n-1}\\ \;+\;0.468\;*\;\Delta RNFLTRate_{n}+ɛ\\ \Delta RNFLTRate_{n}=-0.004-0.353\\ \;*\;\Delta RNFLTRate_{n-1}+ɛ \end{array} \end{array}$$

### Primary Analysis: Does MRW Change Predict RNFLT Change?

The measures of goodness of fit indicated that Model C provide a better fit for the data (RMSEA = 0.101 [CI = 0.089, 0.113] for both), compared to the Model D (previous MRWRate predicts RNFLTRate) (RMSEA = 0.114 [CI = 0.102, 0.127]). Model C also have higher CFI and TLI scores compared to Model D (see Table 3). There is no overlap in the RMSEA CI between Model C (current MRWRate predicts RNFLTRate) and Model D (previous MRWRate predicts RNFLTRate), indicating that Model C yields a statistically significantly better fit.

Similarly, using Model C we found that *ΔRNFLTRate_n-1_* and *ΔMRWRate_n_* were significant predictors of *ΔRNFLTRate_n_* (both *P* ≤ 0.001), which as before means that the previous rate of RNFLT and the concurrent rate of MRW significantly predicts the current rate of RNFLT. We did not find evidence that *ΔMRWRate_n-1_* significantly predicts *ΔRNFLTRate_n_*. Hence, no evidence of a clinically significant time lag for MRW parameter was determined. The coefficient estimates and intercepts that best fit Model C are as follows:

#### Model C: Current MRWRate Predicts RNFLTRate

$$\begin{array}{c} \begin{array}{c} \Delta MRWRate_{n}=-0.009-0.326\\ \;*\;\Delta MRWRate_{n-1}+ɛ\\ \Delta RNFLTRate_{n}=-0.004-0.343\\ \;\;*\;\Delta RNFLTRate_{n-1}+0.079\\ \;\;*\;\Delta MRWRate_{n}+ɛ \end{array} \end{array}$$

### Secondary Analysis: SEM Robustness

To further assess the four models, we fit Models A to D using a greater number of rates per eye (up to 10, opposed to up to four; so using more data, but at the expense of having to assume that the “true” rate of change is constant over a longer period) and using complete data for five visits with no missing visits (n = 354 eyes, opposed to n = 568 eyes). We also fit time lag Models B and D using a time lag of two visits (one year [±3 months] interval opposed to six months [±3 months]). The goodness of fit metrics overall were worse compared to the original models, with higher RMSEA and lower CFI and TLI (see Table 4 for details). When using 10 rates in the four models, RMSEA was found to be marginally smaller (RMSEA = 0.082–0.091) compared to the original models tested (RMSEA = 0.101–0.114), yet the models were not significantly different from each. This was deemed due to the larger number of visits included. However, the CFI (0.274–0.396) and TLI (0.37–0.481) were a lot smaller compared to the original models tested (CFI = 0.535–0.629; TLI = 0.617–0.703, respectively). See Table 4 for details.

**Table 4.** Goodness-of-Fit Measures for the Comparative Models

SEMs	N Eyes	RMSEA		CFI	TLI
Ten Rates	Model A	568	0.083	0.394	0.479
	(current RNFLTRate predicts MRWRate)		(CI, 0.078–0.088)		
	Model B	568	0.090	0.286	0.383
	(previous RNFLTRate predicts MRWRate)		(CI, 0.085–0.095)		
	Model C	568	0.082	0.396	0.480
	(current MRWRate predicts RNFLTRate)		(CI, 0.078–0087)		
	Model D	568	0.091	0.274	0.370
	(previous MRWRate predicts RNFLTRate)		(CI, 0.086–0.096)		
No Missing Data	Model A	354	0.115	0.578	0.662
	(current RNFLTRate predicts MRWRate)		(CI, 0.099–0.130)		
	Model B	354	0.121	0.542	0.623
	(previous RNFLTRate predicts MRWRate)		(CI, 0.106–0.137)		
	Model C	354	0.114	0.582	0.665
	(current MRWRate predicts RNFLTRate)		(CI, 0.099–0.130)		
	Model D	354	0.121	0.544	0.624
	(previous MRWRate predicts RNFLTRate)		(CI, 0.105–0.137)		
Two Time Intervals	Model A	568	0.101	0.628	0.703
	(current RNFLTRate predicts MRWRate)		(CI, 0.089–0.113)		
	Model B	568	0.127	0.443	0.527
	(previous RNFLTRate predicts MRWRate)		(CI, 0.11–0.139)		
	Model C	568	0.101	0.629	0.703
	(current MRWRate predicts RNFLTRate)		(CI, 0.089–0.113)		
	Model D	568	0.125	0.456	0.538
	(previous MRWRate predicts RNFLTRate)		(CI, 0.113–0.138)		

### Secondary Analysis: SEM in Subgroups

To assess whether the conclusions were dependent on disease severity, we split the cohort into two groups and repeated the fitting of Models A–D in each: the subset of “normal” eyes categorized as within normal limits for all three measures (MRW, RNFLT, and MD from automated perimetry), and the remaining cohort categorized as being outside normal limits on at least one of those measures. The goodness of fit metrics overall were slightly better for the RMSEA compared to the original models but were worse for CFI and TLI (see Table 5 for details). This difference is suspected to be due to smaller sample size and range of observations used in these subanalyses. No substantial differences in coefficients were seen when comparing these two subgroups. Overall, the subgroup analyses did not yield a model with better fit than the original model, nor did the trend of the coefficient estimates change. In other words, the lack of evidence of a time lag between MRW and RNFLT in our primary analysis was not just because the eyes we included were not sufficiently early in the disease process.

**Table 5.** Goodness-of-Fit Measures for the Comparative Models for Subgroup Analysis

SEMs		N Eyes	β	*P*	RMSEA	CFI	TLI
Normal	Model A	205	0.297	**<0.001**	0.128	0.570	0.656
	(current RNFLTRate predicts MRWRate)				(CI, 0.108–0.149)		
	Model B	205	0.002	0.980	0.133	0.553	0.632
	(previous RNFLTRate predicts MRWRate)				(CI, 0.112–0.154)		
	Model C	205	0.094	**<0.001**	0.126	0.588	0.670
	(current MRWRate predicts RNFLTRate)				(CI, 0.105–0.147)		
	Model D	205	−0.036	0.077	0.131	0.564	0.641
	(previous MRWRate predicts RNFLTRate)				(CI, 0.110–0.152)		
Abnormal	Model A	363	0.527	**<0.001**	0.112	0.473	0.578
	(current RNFLTRate predicts MRWRate)				(CI, 0.097–0.128)		
	Model B	363	0.107	0.203	0.126	0.355	0.469
	(previous RNFLTRate predicts MRWRate)				(CI, 0.111–0.142)		
	Model C	363	0.074	**<0.001**	0.112	0.473	0.579
	(current MRWRate predicts RNFLTRate)				(CI, 0.097–0.128)		
	Model D	363	0.005	0.676	0.127	0.350	0.465
	(previous MRWRate predicts RNFLTRate)				(CI, 0.112–0.142)		
Older	Model A	288	0.51	**<0.001**	0.093	0.641	0.713
	(current RNFLTRate predicts MRWRate)				(CI, 0.760–0.111)		
	Model B	288	0.113	0.106	0.109	0.525	0.609
	(previous RNFLTRate predicts MRWRate)				(CI, 0.0910–0.270)		
	Model C	288	0.089	**<0.001**	0.09	0.668	0.735
	(current MRWRate predicts RNFLTRate)				(CI, 0.072–0.108)		
	Model D	288	−0.014	0.321	0.110	0.518	0.603
	(previous MRWRate predicts RNFLTRate)				(CI, 0.092–0.128)		
Younger	Model A	280	0.384	**<0.001**	0.137	0.358	0.486
	(current RNFLTRate predicts MRWRate)				(CI, 0.120–0.155)		
	Model B	280	0.005	0.946	0.146	0.292	0.417
	(previous RNFLTRate predicts MRWRate)				(CI, 0.129–0.164)		
	Model C	280	0.069	**<0.001**	0.138	0.350	0.480
	(current MRWRate predicts RNFLTRate)				(CI, 0.121–0.155)		
	Model D	280	0.005	0.752	0.146	0.293	0.418
	(previous MRWRate predicts RNFLTRate)				(CI, 0.129–0.164)		

“Normal” refers to eyes rated functionally or structurally within normal limits, whereas “Abnormal” refers to eyes outside of these limits.

Bold values indicates statistical significance.

To assess whether the conclusions were age dependent, we split the cohort into two groups aged <65 years and aged ≥65 years. Models A–D were repeated in each group. The goodness of fit metrics overall were similar or slightly worse when compared to the original models (see Table 5 for details). These small differences are suspected to be due to smaller sample size and range of observations used in these subanalyses. Overall, no substantial differences in coefficients were seen when comparing these two subgroups. Overall, the subgroup analyses did not yield a model with better fit than the original model, nor did the trend of the coefficient estimates change. Therefore the lack of evidence of a time lag between MRW and RNFLT in our primary analysis was not the result of an age effect.

## Discussion

In this study, we used SEMs to assess whether more severe rates of change in MRW were associated with and potentially predict correspondingly more severe rates of change in RNFLT in eyes with glaucoma and glaucoma suspects. Beyond the immediate clinical implications of earlier detection and glaucoma risk stratification, a comprehensive understanding of the temporal connection between MRW and RNFLT may provide valuable insights into the underlying mechanisms of glaucoma pathophysiology. Yet, we did not find evidence of a clinically significant time lag (i.e., greater than six months to ensure it is clinically useful) for either MRW nor RNFLT. This suggests that even though change might be detected sooner using one measure than the other, there is no predictable causative link whereby change in MRW consistently precedes similar changes in RNFLT or vice versa.

Our primary analysis used series of up to five visits, over approximately a two-year period. We validated the SEMs further by fitting the models using longer series, excluding series with missing values, and increasing the time interval from six months to one year. Nonetheless, the conclusions remained unchanged, and the original models were rated as better fitting the data. To ensure the lack of significant time lag was also the case for glaucoma suspects (i.e., people with early signs of glaucomatous damage for whom MRW has been reported to show detectable change earlier than RNFLT^3^), we repeated the SEMs using data from eyes rated functionally or structurally within normal limits. Still, no clinically significant time lag for either MRW nor RNFLT was identified. This data was compared to our remaining cohort of patients outside these normal limits, and no significant difference in model coefficients was identified.

These findings from a large cohort of people with glaucoma may appear to contrast to the existing literature which has suggested that MRW changes prior to RNFLT in glaucoma. The mechanical influence on axons as a result of increased ONH cupping (due to elevated IOP) likely causes MRW thinning.^6^ As the RNFLT parameter we measured is further away from this region (6° from the BMO centroid), and thus is less influenced by direct effects of IOP,^7^^,^^8^ it follows that changes in MRW would be expected to occur *before* changes in RNFLT. Indeed, He and colleagues identified changes in MRW preceded that of RNFLT in an experimental glaucoma model, therefore concluding a time lag in MRW to be evident in nonhuman primates.^12^ Furthermore, Chauhan et al.^3^ reported that MRW was found to have earlier detectable change compared to RNFLT parameters in early-stage glaucoma.

There are several conceivable reasons to explain our findings. First, glaucoma is more common in older patients (the mean age of our cohort was 63 years [±SD 10]), and evidence suggests that tissues found in the ONH region such as the sclera and lamina cribrosa stiffen significantly as a function of age.^25^^,^^26^ Consequently, there is less susceptibility to mechanical deformation at the ONH.^27^ This may result in MRW thinning concurrently to RNFLT as seen in this study or that the structural damage for the two parameters has a similar onset.

Transient changes in cupping of the ONH occur as a result of fluctuating IOP levels^28^ and cannot be feasibly controlled for in human experimental studies.^28^^,^^29^ Therefore any IOP measures or indeed parameters impacted by IOP changes such as MRW, are a mere snapshot of these fluctuating changes and may not reflect the more chronic structural glaucomatous changes in the eye. Conversely, in experimental glaucoma studies, it is possible (and standard practice) to stabilize IOP to reliably assess OCT metrics. Thus this difference in experimental procedure may explain why He and colleagues^12^ found a time lag, whereas this study did not. Further elaborating on the impact of transient cupping as a result of IOP changes, not only is it known that fluctuating IOP and susceptibility to IOP-related damage varies across individuals,^30^ but the cohort in the P3 study are managed clinically and are therefore in receipt of medication aimed to reduce IOP. Thus the MRW parameters in this study can be expected to be more variable, potentially obscuring any true time lag.^11^

Third, it cannot be ruled out that the hypothesized six-month time lag between MRW and RNFLT may be the wrong time interval. To test whether a longer time interval was required to determine a time lag, we validated our SEMs by assessing a time interval of one year (± three months), yet this did not yield any change in the trend of data, and again no time lag was found. A time lag of much less than six months remains consistent with our results but would be of less translational interest because glaucoma suspects are typically only seen in clinics once or at most twice per year,^31^ so such a time lag would be too short to be relevant clinically.

Last, it remains possible that MRW does indeed change earlier than RNFLT as glaucoma develops, as suggested by previous literature.^3^^,^^12^ In this scenario, the first stage would consist of both conformational and remodeling-based changes at the ONH that alter MRW but not RNFLT; followed by a second stage at which axon loss occurs and both MRW and RNFLT change concurrently. Our results then suggest that the transition between those stages occurs relatively abruptly, rather than a gradual transition over several years, which would be detectable as a time lag in our models.

Use of the SEM framework in this study opposed to simpler statistical techniques enabled us the advantage of simultaneously using a single variable as both an outcome and a predictor, necessary for this type of comparison. SEMs are also amenable to assessing time lags across longitudinal data, allowing for a more comprehensive analysis of the temporal relationships between MRW and RNFLT. Furthermore, SEMs provides methods for dealing with data missingness, which is advantageous when dealing with longitudinal data of this nature, where the propensity for missed visits can be frequent. Last, there are various model fit indexes available to the lavaan package in R (used to build and test SEMs), which evaluate the goodness of fit between the model and the data. This aided the study in ensuring that the best model was chosen that represented the observed data. A disadvantage of SEMs is that their use requires that longitudinal data have consistent inter-visit time intervals. In our dataset, the mean inter-visit interval in the absence of a missed visit was 6.2 months, with an SD ±23 days and a range of 118 to 267 days. The SEM technique would be inappropriate for larger datasets taken directly from routine clinical practice, where the inter-visit interval is not just much more variable but is also directly related to the assessed likelihood of significant disease progression. Notably, the more simplistic correlation analysis yielded the same conclusion that there was no evidence of a significant time lag.

Additional strengths of this study include the large dataset of 568 eyes of 287 people with glaucoma or suspected glaucoma, followed longitudinally as part of the P3 study. The battery of standardized testing completed in the P3 study since 2009 gave a wealth of both structural (OCT) and functional (SAP) data to be compared over a long time period. Thus the data are well controlled and of good quality (OCT scan quality score >15), with regularly timed visits, and is expected to be of higher quality compared to that sourced from electronic health records.

A caveat of this study is that the cohort primarily consists of white individuals, which may restrict the generalizability of the findings, because ethnicity-based differences in glaucoma have been observed and may not be fully represented within this predominantly homogenous population. The P3 dataset lacks a substantial number of patients with late-stage glaucoma. Although our analysis revealed no significant differences between eyes within structural and functional normal limits and those outside these limits, it remains plausible that results may be different in severe glaucoma. We also note that even if the average rates of change of MRW or RNFLT vary with severity, age, or both, this would not necessarily cause any change in the time lag between them. Further investigation is warranted to explore potential differences in the rate of change in patients with advanced glaucoma. Furthermore, although MRW provides valuable information, it may not fully capture all structural changes within the ONH and anterior lamina cribrosa surfaces.^32^ In light of this consideration, future research should explore lamina cribrosa displacement to achieve a more comprehensive understanding of optic nerve head deformation in the context of glaucoma. Last, it is important to note that factors potentially influencing the rate of change in MRW, including IOP and the use of ocular hypertensive medications, were not explored as covariates in our study. However, preliminary analysis did not reveal a significant correlation between IOP and the rate of change in MRW and RNFLT in this treated cohort. Additionally, we assume that the primary effect of ocular hypertensive medication would be to lower IOP, contingent on patient adherence and, hence, that they are unlikely to affect the time lag between different measurements. Due to these considerations, incorporating these factors as covariates in our models was deemed unfeasible.

## Conclusions

In summary, a clinically significant time lag in MRW compared to RNFLT was not identified in a large cohort of people with glaucoma. That is, the rate of change of MRW over a six-month period did not predict the rate of change of RNFLT over the subsequent six months or vice versa. As RNFLT is less variable to assess (due to being less impacted by IOP fluctuations), it is suggested that this parameter is of more use in the early assessment of glaucomatous progression.

## Supplementary Material

Supplement 1

Supplement 2
