# Supplementary material for: The Temporal Relation Between Rates of Retinal Nerve Fiber Layer and Minimum Rim Width Changes in Glaucoma
Source: Transl Vis Sci Technol. 2024 Apr 5;13(4):10. doi: 10.1167/tvst.13.4.10 (PMC11005071; doi:10.1167/tvst.13.4.10)
Supplement: Supplement 1 [file tvst-13-4-10_s001.pdf]

Table S1. Demographic Characteristics of Participants in Subgroup Analysis. Data sourced from self-report.

Demographics	Cohorts of Participants (%)			
	Normal	Abnormal	Older (>= 65 years)	Younger (< 65 years)
**N (N Eyes)**	136 (205)	217 (363)	146 (288)	141 (280)
**Gender**				
Male	56	81	58	53
Female	80	136	88	88
**Ethnicity**				
White	131	191	137	122
Black	2	6	3	4
Asian	0	12	5	7
Mixed	1	4	1	3
Native American	1	2	0	2
Unknown	1	2	0	3

Note: 'Normal' refers to eyes rated functionally and/or structurally within normal limits, while 'Abnormal' refers to eyes outside of these limits. 568 eyes are split into either Normal or Abnormal, meaning one patient could have eyes in either cohort and their demographic data would be included in both cohorts.
