# Supplementary material for: The Temporal Relation Between Rates of Retinal Nerve Fiber Layer and Minimum Rim Width Changes in Glaucoma
Source: Transl Vis Sci Technol. 2024 Apr 5;13(4):10. doi: 10.1167/tvst.13.4.10 (PMC11005071; doi:10.1167/tvst.13.4.10)
Supplement: Supplement 2 [file tvst-13-4-10_s002.pdf]

Table S2. Baseline Characteristics of Eyes in Subgroup Analysis.

	Mean	Standard Deviation	Range
**Age (years)**			
Normal	62	11	33, 85
Abnormal	65	9	39, 86
Older*	72	5.57	65, 86
Younger*	56	6.85	33, 64
**Mean Deviation (dB)**			
Normal	0.49	1.21	-3.26, 6.72
Abnormal	-1.94	3.42	-17.69, 2.81
Older	-1.25	3.18	-17.69, 3.03
Younger	-0.58	2.69	-15.46, 6.72
**Retinal Nerve Fiber Thickness (μm)**			
Normal	95.20	7.89	78.77, 114.67
Abnormal	80.03	15.59	38.78, 121
Older	85.11	15.70	39.95, 121
Younger	85.93	14.66	38.78, 120
**Minimum Rim Width (μm)**			
Normal	302.28	44.49	227.7, 401.1
Abnormal	234.55	64.27	79.5, 485.5
Older	253.21	63.43	99.7, 485.5
Younger	265.01	68.91	79.5, 442.3
**Intraocular Pressure (IOP)**			
Normal	17.82	3.24	8, 26
Abnormal	16.71	3.97	7, 29
Older	16.83	3.7	7, 29
Younger	17.54	3.69	8, 27

Note: 'Normal' refers to eyes rated functionally and/or structurally within normal limits, while 'Abnormal' refers to eyes outside of these limits. Older cohort aged >= 65 years; Younger cohort aged < 65 years. The normal and abnormal subgroups were based on the average of the first five visits being within or outside normative limits. This table shows the baseline visit data.
